# Death of Dr. Taylor

**Published:** 1878-08-20

**Authors:** 


### DEATH OF DR. TAYLOR.

We regret to learn that Dr. J. N. Taylor, of Cotile, Louisiana, is dead.

